# Balancing Complexity and Performance in Convolutional Neural Network Models for QUIC Traffic Classification

**DOI:** 10.3390/s25154576

**Published:** 2025-07-24

**Authors:** Giovanni Pettorru, Matteo Flumini, Marco Martalò

**Affiliations:** 1 Department of Electrical and Electronic Engineering, University of Cagliari, 09123 Cagliari, Italy; giovanni.pettorru@unica.it; 2 Research Unit of Cagliari, National Inter-University Consortium for Telecommunications (CNIT), 09123 Cagliari, Italy; 3 Adesso Schweiz AG, Vulkanstrasse 106, 8048 Zurich, Switzerland

**Keywords:** network traffic classification (NTC), QUIC, convolutional neural network (CNN), deep learning (DL), 6G

## Abstract

The upcoming deployment of sixth-generation (6G) wireless networks promises to significantly outperform 5G in terms of data rates, spectral efficiency, device densities, and, most importantly, latency and security. To cope with the increasingly complex network traffic, Network Traffic Classification (NTC) will be essential to ensure the high performance and security of a network, which is necessary for advanced applications. This is particularly relevant in the Internet of Things (IoT), where resource-constrained platforms at the edge must manage tasks like traffic analysis and threat detection. In this context, balancing classification accuracy with computational efficiency is key to enabling practical, real-world deployments. Traditional payload-based and packet inspection methods are based on the identification of relevant patterns and fields in the packet content. However, such methods are nowadays limited by the rise of encrypted communications. To this end, the research community has turned its attention to statistical analysis and Machine Learning (ML). In particular, Convolutional Neural Networks (CNNs) are gaining momentum in the research community for ML-based NTC leveraging statistical analysis of flow characteristics. Therefore, this paper addresses CNN-based NTC in the presence of encrypted communications generated by the rising Quick UDP Internet Connections (QUIC) protocol. Different models are presented, and their performance is assessed to show the trade-off between classification accuracy and CNN complexity. In particular, our results show that even simple and low-complexity CNN architectures can achieve almost 92% accuracy with a very low-complexity architecture when compared to baseline architectures documented in the existing literature.

## 1. Introduction

The upcoming deployment of sixth-generation (6G) wireless networks promises to significantly outperform 5G in terms of data rates, spectral efficiency, device densities, and, most importantly, latency and security [1]. To cope with the increasingly complex network traffic, Edge Computing will be pivotal, enabling low-latency processing and efficient distribution of computational load [2]. This becomes especially crucial in the Internet of Things (IoT) domains, such as Smart Cities, eHealth, and Industrial IoT, where real-time responsiveness and security are key. IoT-compliant platforms, particularly low-cost, energy-efficient single-board computers, will play an essential role in these edge environments, handling tasks from traffic management to security enforcement [3]. However, achieving an optimal balance between classification accuracy and computational efficiency is vital for ensuring these systems can operate effectively in real-world scenarios [4].

Network Traffic Classification (NTC) works by intercepting packets, extracting detailed statistical and/or behavioral attributes from the traffic, and then feeding this information to appropriate classification algorithms [5]. NTC generally uses three basic approaches: port-based, payload-based, and Machine Learning (ML)-based approaches [6]. Port-based techniques associate services with registered port numbers and classify traffic accordingly. Despite their simplicity, they face challenges from emerging communication methods that ensure private and dynamic port usage [7]. Payload-based methods analyze packet payloads for feature extraction [8] but encounter difficulties in the modern Internet, where encrypted communications are experiencing significant growth, as evident from Google transparency reports [9]. In response, ML-based methods have emerged as a solution for handling both encrypted and unencrypted traffic [10].

Traditional ML techniques, e.g., k-Nearest Neighbor (k-NN) or random forest (RF), have limited traffic classification accuracy if used alone due to their inherent structure. These models rely on predefined, superficial feature extraction methods and are not capable of capturing the complex, non-linear relationships and sequential or temporal patterns in traffic flows within encrypted traffic, which leads to reduced performance in such contexts [10]. To overcome these limitations, deep learning (DL) methods, such as Convolutional Neural Network (CNN) algorithms, have been introduced [11]. A comprehensive literature review demonstrates the interest of the academic community in CNN-based NTC in the presence of encrypted traffic. The authors of [12] present a method for classifying encrypted traffic using a one-dimensional CNN based on reconstructing the traffic from the first 500 bytes of the payload and a length threshold identifier in the header. In addition, CNN, augmented with a Multi-Layer Perceptron (MLP), is proposed in [13] to classify traffic by extracting and analyzing the Server Name Indication (SNI) attribute. Specifically focusing on network security issues, NTC using a 2D-CNN model is introduced in [14], demonstrating high accuracy in identifying both regular and malicious encrypted traffic. A common aspect of these papers is the use of datasets of conventional encrypted traffic, such as HTTPS-based or tunnelled through VPN [15,16].

**Figure 1:**
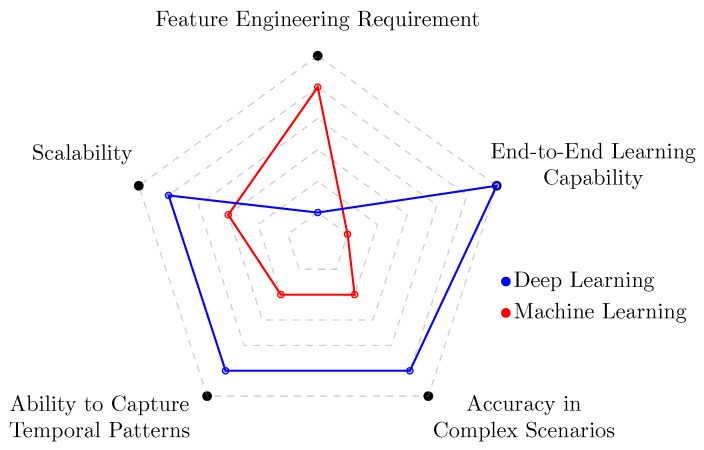
Comparison of DL and traditional ML techniques across key performance dimensions for encrypted traffic classification.

To visually summarize the comparative advantages of DL in this context, Figure 1 presents a radar plot that contrasts DL with traditional ML techniques across five critical dimensions relevant to encrypted traffic classification: feature engineering requirement, scalability, temporal pattern modeling, classification accuracy, and end-to-end learning capability [10].

### 1.1. Related Work on QUIC

A statistical analysis of the literature reveals the increasing use of the encrypted QUIC protocol, developed by Google and currently being standardized by the Internet Engineering Task Force (IETF) [17]. The integration of QUIC-based communication protocols across a diverse array of services in next-generation 6G-enabled networks is anticipated to be a key focus for the research community [18,19]. By seamlessly integrating the efficiency and low latency of User Datagram Protocol (UDP) with the robust security of Transport Layer Security (TLS), along with several functional mechanisms, QUIC is gaining popularity among network communication protocols [20]. Nowadays, over 50% of connections from Chrome browsers to Google servers are made using QUIC [21]. Originally designed to optimize web browsing by improving latency and security, QUIC is now expanding beyond its initial scope to support a broader range of applications, including Internet of Things (IoT) communications. While its adoption has been driven mainly by web browsing and online services, QUIC is now expanding beyond its initial scope to support a broader range of applications, including Internet of Things (IoT) communications [22]. Its low latency, connection migration capabilities, and built-in encryption make it a promising candidate for secure and efficient IoT networking [23].

### 1.2. Main Contribution

Building upon the context and challenges outlined in the previous section, this paper addresses CNN-based NTC for QUIC-encrypted communications, with a specific focus on the trade-off between classification accuracy and model complexity. In particular, it offers the following key contributions.

- Recognizing the limited research on QUIC traffic despite its growing adoption, particularly within IoT scenarios, our study evaluates various CNN-based architectures for NTC of QUIC-encrypted communications;
- In contrast to prior studies that prioritize high accuracy, e.g., [24,25,26,27], our objective is to explore and demonstrate the trade-off between classification accuracy and model complexity, which is an essential factor for deployment on resource-constrained edge devices;
- We extend the results of previous works [27] by proposing a simple CNN-based architecture with minimal computational requirements, achieving good accuracy (nearly 92%) while being suitable for real-world implementation.

## 2. Reference Scenario

**Figure 2:**
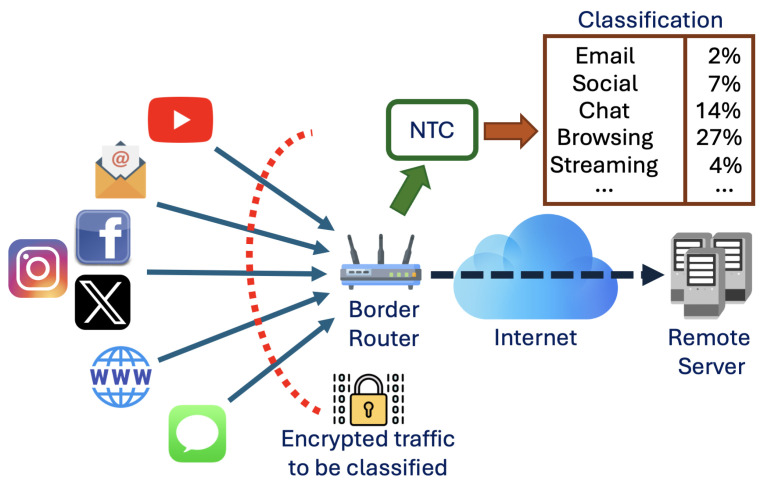
Reference scenario.

Figure 2 illustrates the reference scenario where users connect to a remote server over the Internet to access services such as web browsing, video streaming, and email. Encrypted data from these services is transmitted through the network and collected by the border router for traffic analysis. In this paper, we focus on QUIC, leveraging the recent adoption of this protocol for low-latency and encrypted communications [17].

The traffic classification process is outlined by a sequential scheme that can be summarized as follows [28]:

1. *Packet collection*: In the initial stage, the router gathers a (possibly huge) amount of packets from diverse users and services on the network;
2. *Feature extraction*: The collected packets are analyzed to extract relevant features that allow for distinguishing the different traffic classes;
3. *Classification*: The traffic classes are identified using a classification algorithm.

Regarding the latter point, different methods can be used, either based on simple comparison operations (e.g., port-based classification) or using a trained model, as in the case of statistical- and ML-based classifiers [29]. In particular, our approach leverages an ML algorithm based on the adoption of CNNs, as detailed in Section 3.

## 3. Network Traffic Classification Scheme

In this section, we present the baseline setup and design of the CNN-based NTC models employed in the experimental phase of this work, detailing the architectural choices and the rationale behind them. We progressively analyze each model configuration, highlighting structural simplifications that form the core of our trade-off discussion between accuracy and complexity. Finally, we describe the set of traffic flow features that enable our models to effectively classify encrypted QUIC-based traffic.

### 3.1. Baseline Setup

**Figure 3:**
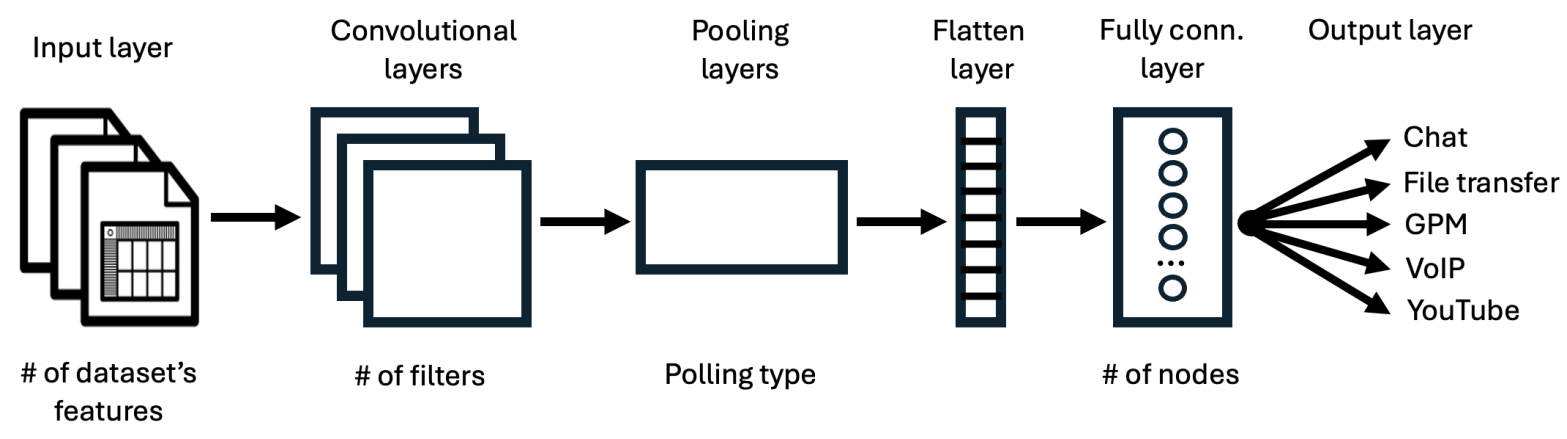
General NTC architecture.

For our NTC models, we employed a DL strategy, specifically utilizing CNNs. To strike a balance between computational complexity and accuracy, we developed multiple models with varied architectures, either conventional or based on an Artificial Neural Network (ANN). The general NTC architecture is illustrated in Figure 3 and is composed of the following layers [30]:

- *Input layer*, which receives input data and passes it to the CNN.
- *Convolutional layer* (one or more), which performs a convolution operation on the input data using trainable filters to create a feature map that encapsulates the various features present in the input data.
- *Pooling layer* (one or more), which reduces the spatial dimensions of the feature maps generated by the convolutional layer, thus allowing the capture of prominent features while reducing computational complexity. In this category, we include Max Pooling and the Dropout layer.
- *Flatten layer*, which reshapes the output of previous layers into a one-dimensional array to be input in the following stage.
- *Fully Connected layer* (one or more), in which neurons are connected to every activation in the previous layer, enabling high-level feature learning.
- *Output layer*, which produces the final model’s predictions or outputs, i.e., the traffic classes.

These architectures vary in the number of layers, parameters, and complexity, as described in the following sections. During model construction, critical decisions are made, including the selection of optimization algorithms to minimize the loss or cost function. Several first-order optimization algorithms, including Adam, SGD, RMSprop, AdamW, Adadelta, Adagrad, Adamax, and Nadam have been considered and implemented with Keras 2.11, an open-source, deep learning framework written in Python (available at https://keras.io). To identify the most suitable optimizer, the dataset was split into 80% for training and 20% for testing. Each model was then trained for 20 epochs, with validation performed at each epoch by reserving 15% of the training data for validation purposes. After training, the models were evaluated not only in terms of accuracy but also using additional metrics of interest, including precision, recall, and F1-score. The results obtained from these experiments, which are not included as they fall outside the main scope of the paper, highlight that Adam is the most suitable optimizer due to its computational efficiency, small memory footprint, robustness to gradient scaling, and suitability for large datasets and parameters, as described in [31].

### 3.2. Proposed Models

In the course of this study, numerous implementations of the considered CNN-based NTC architecture in Figure 3 have been tested, with a particular focus on analyzing the architectural composition and its influence on model performance and complexity.

For each architectural variant, once compiled and configured, the model was trained using 80% of the dataset, with 15% of this portion reserved for validation. Leveraging Keras’ built-in training routines, each model was trained over 20 epochs using batches of 32 samples (totaling 4909 batches), allowing us to monitor training and validation loss and accuracy at the end of each epoch. These metrics were then exploited by the optimizer to progressively refine the model’s performance across epochs. Through an iterative process of trials and refinements, we identified the best-performing configurations, which are summarized in Table 1. These architectures are presented in detail in the remainder of this section to highlight the structural choices behind each model, an essential aspect for understanding the rationale of our work and the trade-offs between accuracy and computational efficiency.

*Model #1*—The first architecture, the only ANN-based model among those of interest, is made up of three Fully Connected layers, each with an output space of 512 units and ReLU [32] activation functions. These layers are followed by a Dropout layer with a rate of 0.1 to mitigate potential overfitting concerns. A Flatten layer is then introduced to convert the multidimensional data from the previous layers into a single vector to facilitate further processing. This preprocessing step sets the stage for two additional Fully Connected layers that mirror the characteristics of the first three layers, along with three others with an output space of 256 units each. Finally, a Fully Connected output layer with a Softmax [33] activation function and an output space of 5 to match the number of classes completes the architecture. This initial network, which is a feed-forward type, is composed entirely of typical Fully Connected layers, reminiscent of a basic neural network. Its creation serves as a benchmark for comparison with subsequent CNN models, all of which are expected to overcome its performance.

*Model #2*—This architecture includes two initial Convolutional layers, each with 128 filters of size 2 × 2, using ReLU activation functions. Padding is applied after convolution to maintain the original dimensions. These layers are followed by a Max Pooling layer. Next comes another Convolutional layer with 64 filters, followed by another Max Pooling layer. A Flatten layer then transforms the multidimensional data from the previous layers into a single vector, preparing it for the following four Fully Connected layers, each with an output space of 512. Finally, the architecture includes a Fully Connected output layer using a Softmax activation function and an output space of 5 to match the number of classes. The network follows a feed-forward structure, with an initial CNN-like section consisting of Convolutional and Pooling layers, followed by Fully Connected layers.

*Model #3*—The next architecture consists of four initial Convolutional layers with filter counts of 512, 256, 128, and 128, each 2 × 2 in size, using ReLU activation functions. A Dropout layer with a rate of 0.1 is then introduced to mitigate potential overfitting problems. Next, another set of four Convolutional layers with 128 filters each, also 2 × 2 in size, and ReLU activation is added. This is followed by a Max Pooling layer and a Flatten layer, which converts the multidimensional data from the previous layers into a single vector. A Fully Connected layer with an output space of 512 is then linked to the Flatten layer, which, in turn, is coupled to the final Fully Connected layer, which serves as the output layer. This layer has an output space of 5, corresponding to the number of classes, and uses Softmax activation. The CNN-based structure is of the feed-forward type.

*Model #4*—The following architecture represents a significant simplification from the previous one. It consists of four initial Convolutional layers of 512, 256, 128, and 128 filters, respectively, each of size 2 × 2. ReLU activation functions follow each Convolutional layer. These are followed by a Dropout layer with a rate of 0.1 to mitigate potential overfitting. After the Dropout layer, only two Convolutional layers are used, each with 128 filters of size 2 × 2, again with ReLU activation functions. Next, a Max Pooling layer and a Flatten layer transform the multidimensional data into a single vector. The Flatten layer is linked to a 512-unit Fully Connected layer, which is directly coupled to the final 5-unit Fully Connected layer, which serves as the output layer and reflects the classes to be detected. The output layer uses the Softmax activation function. Like the previous architecture, it is a feed-forward CNN.

*Model #5*—The final architecture presented represents a further simplification over previous solutions. Instead of the classical four convolutional layers, it has only three. These layers have 512, 256, and 128 filters, respectively, each 2 × 2 in size, and use ReLU as the activation function. These layers are followed by a Dropout layer with a rate of 0.1 to mitigate overfitting problems. Next, two Convolutional layers with 128 filters of size 2 × 2 and ReLU activation functions are added. A Max Pooling and a Flatten layer are then applied to convert the multidimensional data into a single vector. This vector is then linked to a Fully Connected layer of 512 units, which is directly coupled to the final Fully Connected output layer of 5 units, corresponding to the number of classes to be detected. The output layer uses Softmax activation. Similar to previous architectures, this network is a feed-forward CNN.

*Model #6*—In this architecture, the previously implemented simplifications are further refined. It consists of two initial Convolutional layers with 128 and 64 filters of size 2 × 2, respectively, both using ReLU activation functions. Following these layers is a Dropout layer with a rate of 0.1, aimed at mitigating overfitting. Instead of four, there are two subsequent Convolutional layers, each with 32 filters of size 2 × 2 and employing ReLU activation. A Max Pooling layer and a Flatten layer follow suit. The latter connects to a Fully Connected layer with an output space of 128, which, in turn, is directly linked to the final Fully Connected layer. This last layer, serving as the output layer, has an output space of 5, representing the number of classes to be recognized, and utilizes Softmax activation. The network follows a feed-forward architecture and, like its predecessor, is a CNN.

*Model #7*—The last architecture consists of two initial Convolutional layers, each with 64 filters of size 2 × 2, both utilizing ReLU activation functions. Following these layers is the standard Dropout layer with a rate of 0.1. Subsequently, there are two Convolutional layers, each with 32 filters of size 2 × 2, still employing ReLU activation. A Max Pooling layer and a Flatten layer ensue. The Flatten layer connects to a Fully Connected layer with an output space further reduced to 64, which then links directly to the final Fully Connected layer. This output layer has an output space of 5, representing the number of classes to be recognized, and employs Softmax activation. The network follows a feed-forward architecture and, like the previous one, is a CNN.

Models #6 and #7 are derived from an empirical and iterative simplification strategy applied to the baseline architectures, aimed at progressively reducing overall model complexity by limiting the number and size of convolutional and dense layers, as well as simplifying operations like pooling and dropout, while preserving acceptable classification performance. This approach avoids extensive hyperparameter tuning to focus on evaluating the impact of architectural downsizing, as discussed in Section 4.2.

### 3.3. Traffic Flow Features

Following the discussion in the previous paragraphs, the selected features for classification are intentionally limited to those that remain observable despite encryption. Since QUIC traffic is encrypted by design, payload-based inspection becomes ineffective; as a result, we focus on features derived from metadata and transport-layer information that are not affected by encryption. Starting from the feature set introduced in [27], which serves as a benchmark for this study, we select a subset of seven main flow-related features aimed at reducing computational complexity while preserving classification effectiveness:

- Packet length;
- Time relative elapsed since the first packet;
- Time elapsed since the previous packet;
- Percentage of large packets in a flow;
- Percentage of small packets in a flow;
- Flow size;
- Flow duration.

For the first three features, the mean, standard deviation, skewness, and kurtosis statistical indices are computed. This choice is based on the observation that, even if the features are packet-based, these (possibly high-order) statistical indices give us information on the flow behavior. In particular, the selected quantities have significantly different values for the QUIC traffic classes discussed in Section 4, making it easy for our NTC models to distinguish between them. As a result, each stream is characterized by a comprehensive set of 16 features, including both primary and derived metrics. This holistic feature representation, designed to be robust against encryption and derived from an established benchmark, offers a solid foundation for classification, as evidenced by the good accuracy achieved across different model architectures presented in Section 4.

## 4. Performance Evaluation

This section presents the experimental framework and performance evaluation of the proposed classification models. It begins by describing the reference dataset, preprocessing steps, and class distribution analysis, followed by a detailed discussion of the adopted evaluation metrics and the numerical results obtained. The analysis aims to assess the effectiveness of our solutions while highlighting the trade-offs between model complexity and classification performance in a realistic traffic scenario.

### 4.1. Reference Dataset and Preprocessing

To evaluate the performance of the proposed schemes, we resort to the dataset presented in [27,34]. Since the core focus of this work is on the architectural complexity of the proposed NTC rather than on data representation, readers are referred to [27,34] for detailed information on the data structure, licensing, and collection methodology. The dataset itself consists of Wireshark captures collected over several weeks, amounting to over 100 GB of network traffic. It comprises flows categorized into five different QUIC-based classes of service: Google Hangout Chat, Google Hangout VoIP, Google Play Music (GPM), File Transfer (FT), and YouTube (YT), providing a rich and varied foundation for our classification task.

Python is employed to manage the dataset, using the Pyshark 0.5 library for data import and segmenting traffic into flows by socket addresses and protocol. The features discussed in Section 3.3 are extracted and grouped with the Statistics and Pandas libraries, followed by outlier removal, reducing the dataset to around 230k flows. A further step in the preprocessing phase involves normalizing the dataset using Min-Max scaling, which adjusts all feature values to a range between 0 and 1. This ensures that all features contribute equally to the learning process, preventing those with larger numerical ranges from disproportionately influencing the model. Additionally, the class labels are encoded to enable the correct classification of traffic flows. Since we are addressing a multi-class classification problem using neural networks, the labels are transformed through one-hot encoding, converting categorical string values into a binary format, making them suitable for the model’s output structure.

The preprocessed dataset is notably unbalanced, with VoIP and YT accounting for approximately 31% of the flows, GPM for around 24%, FT for 10%, and Chat for just 4%. This uneven distribution could potentially introduce bias during training, as models may favor the more represented classes while struggling to learn effective decision boundaries for minority classes, such as FT. To address this concern, we performed a detailed per-class analysis, focusing on the distribution and behavioral patterns of each traffic type. The results reveal that, despite the imbalance, each class exhibits distinct and consistent characteristics. For instance, YT flows display a significantly higher average time since the first packet, while the FT class—although limited to fewer than 10,000 samples—demonstrates unique and easily distinguishable features compared to all others. These traits suggest that FT flows may still be reliably recognized, even with limited data. Given the presence of such discriminative patterns across all classes, the entire dataset was retained for training, accepting the imbalance as a realistic representation of traffic distribution.

### 4.2. Numerical Results

The models’ performance is evaluated using a standard processing phase, where 80% of the dataset is used for training and validation and 20% for testing. To ensure independence from dataset portions, we use 10-fold cross-validation, repeating the analysis with different data partitions each time. While detailed, fold-by-fold results fall outside the primary scope of this work, which is focused on exploring performance–complexity trade-offs, we observe limited variation across folds (within 1–2 percentage points) for all metrics, including accuracy, precision, recall, and F1-score. This supports the use of average values as a reliable indicator for comparative analysis.

The following “standard” ML-related performance metrics are evaluated. Considering True Positives (TP), True Negatives (TN), False Positives (FP), and False Negatives (FN) [11], the accuracy *A* can be defined as

(1) $$A=\frac{TP+TN}{TP+FP+FN+TN}.$$

In particular, the accuracy is differentiated between train accuracy ($A_{train}$), validation accuracy ($A_{valid}$), and test accuracy ($A_{test}$). Moreover, precision *P* is defined by how many of the positive predictions made are truly positive, i.e.,

(2) $$P=\frac{TP}{TP+FP}.$$

Then, the recall *R* is defined by how many of the true positives are correctly predicted as positives, i.e.,

(3) $$R=\frac{TP}{TP+FN}$$

Finally, the F1-score *F* is defined as the harmonic mean between *P* and *R* so as to have a more balanced performance, i.e.,

(4) $$F=2\;\frac{P\times R}{P+R}$$

It is worth mentioning that, in the case of the last three metrics (i.e., *P*, *R*, and *F*), the weighted average is computed to take into account the use of an unbalanced dataset.

Table 2 shows average cross-validation performance (in percentage terms) for models 1–5, including fold-by-fold variation ranges.

The main finding is that the best performance is obtained with model #5, which is also the simplest in terms of architectural complexity. Compared to the NTC scheme in [27], our models reach an average overall accuracy *A* of approximately 92%, about 7–8% lower than the benchmark. However, the reference approach achieves its performance through a multi-stage pipeline and the use of hundreds of features, whereas our method relies on a single-stage CNN fed by only 16 features. This considerable reduction in complexity makes our models particularly suitable for deployment in near-real-time and resource-constrained environments, such as IoT edge devices. Whether the observed 92% accuracy represents an acceptable compromise within our study’s focus on balancing accuracy and complexity is inherently dependent on the specific application context. In scenarios where computational efficiency, low latency, or energy constraints are critical, such a trade-off may not only be acceptable but also preferable.

**Figure 4:**
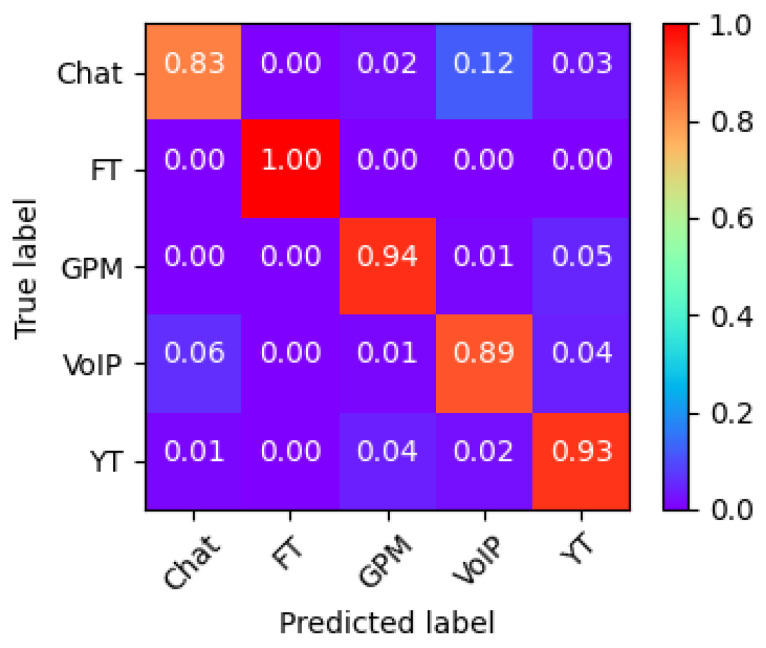
Model #5 confusion matrix.

We now analyze the accuracy confusion matrix of the best-performing model, #5, to identify which class is most critical for accurate label prediction. Such a confusion matrix is shown in Figure 4. First, the FT class achieves near-perfect recognition, approaching 100% accuracy, despite having fewer samples. This is due to the highly distinct characteristics of some features. On the other hand, the worst classification happens with the Chat class, which was often confused with the VoIP class. This is due to their association with the same application, i.e., Google Hangouts, which generates similar traffic patterns for both Chat and Voice services, for example, in terms of the distribution of small packets (less than 150 bytes), resulting in overlapping flow characteristics that hinder accurate classification [27]. Note that removing the Chat class from the dataset significantly improved overall performance; in particular, a 93–95% accuracy can be achieved with such simple models.

**Figure 5:**
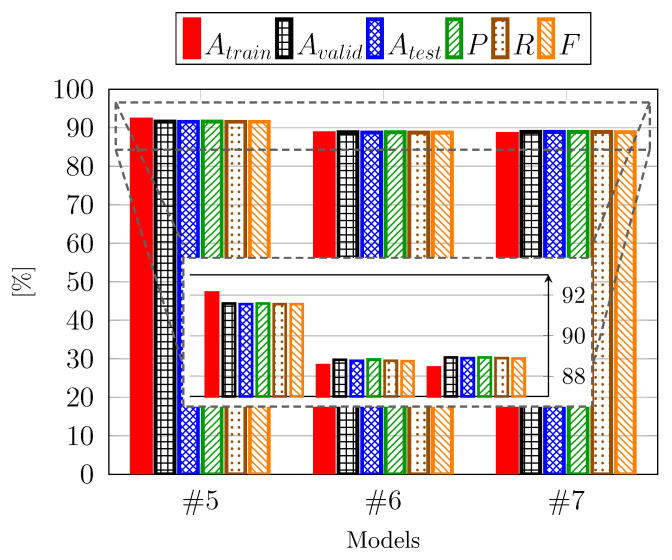
Performance of simplified CNN-based architectures.

As depicted in Table 2, the simplest model emerged as the top performer. To determine if the trend of achieving both simplicity and high performance persisted, we compared two additional models, #6 and #7, with the best-performing model from the previous step, Model #5. These new models feature progressively streamlined architectures, reducing both the number of layers and the complexity of individual layers. In particular, such models present an eight-layer architecture, with Model #7 further simplified with respect to #6 in terms of the parameters and operating characteristics of each layer, making it exceptionally straightforward. Upon conducting the same evaluation steps as with the previous models, the results are illustrated in Figure 5, which includes both the full performance range starting from a zero baseline and a zoomed-in view on the most relevant performance interval. Notably, there was a performance deterioration, falling below 89%, signifying a reversal in the trend. Consequently, it can be concluded that among the first five implemented models, the final model represents the optimal compromise between simplicity and good accuracy. Consistent with the analysis presented in Table 2, the fold-by-fold variation is minimal, even for these models, and thus, we do not report the standard deviation for this figure to maintain clarity and focus on the mean performance trend.

## 5. Conclusions

In the context of emerging 6G networks, where low latency, security, and high efficiency are paramount, the design of scalable and resource-aware NTC models becomes increasingly crucial. This challenge is further heightened by the widespread adoption of Edge Computing and the deployment of IoT-compliant platforms as edge nodes, where achieving an effective balance between security and computational efficiency becomes essential.

In this paper, we have evaluated CNN models for classifying encrypted traffic generated by QUIC-based services using packet flow-based classification. The proposed models leverage ML-based approaches to classify services such as Google Hangout chat, VoIP, file transfer, Google Play Music, and YouTube. Various architectures with different levels of complexity are considered, and their accuracy is evaluated. Compared to the baseline NTC scheme in [27], our approach has a lower overall accuracy of approximately 91%, while the reference model has an accuracy of 99%. However, our model is characterized by its architectural simplicity, employing a single-stage CNN with a limited number of layers and relying on only 16 input features, which is in contrast to the baseline model that combines a random forest with a CNN and leverages hundreds of features. The results underscore that simpler models can deliver robust performance without requiring increased complexity. This finding offers valuable insights for researchers, indicating that simpler architectures may prove more effective and resource-efficient in practical applications, particularly in IoT environments where computational resources are limited.

Our future work aims to further improve the model’s accuracy, particularly in distinguishing Google Hangout chat from VoIP, while preserving, or even enhancing, its low-complexity architecture. Moreover, we plan to extend the proposed approach to protocols beyond QUIC and validate the models through experimental testing on IoT-compliant platforms, such as Raspberry Pi, to assess their real-world performance under constrained computing conditions.

## Figures and Tables

**Table 1:** Characteristics of the various layers in the considered NTC architectures.

Name	Type	Convolutional	Pooling Layer		Flatten	Fully Connect.
			**Max Pool.**	**Dropout**		
#1	ANN	-	-	1	1	8 (5 × 512 + 3 × 256)
#2	CNN	3 (2 × 128 + 64)	2	-	1	4 (4 × 256)
#3	CNN	8 (512 + 256 + 6 × 128)	1	1	1	1 (256)
#4	CNN	6 (512 + 256 + 4 × 128)	1	1	1	1 (256)
#5	CNN	5 (512 + 256 + 3 × 128)	1	1	1	1 (256)
#6	CNN	4 (128 + 64 + 2 × 32)	1	1	1	1 (128)
#7	CNN	4 (2 × 64 + 2 × 32)	1	1	1	1 (64)

**Table 2:** Average cross-validation performance (in percentage terms), including fold-by-fold variation ranges.

Metric	Model				
	#1	#2	#3	#4	#5
$A_{train}$	89.53 (±1.84)	91.68 (±1.47)	91.55 (±1.51)	91.97 (±1.22)	92.12 (±1.19)
$A_{valid}$	90.12 (±1.91)	90.77 (±1.73)	90.79 (±1.69)	91.09 (±1.38)	91.58 (±1.14)
$A_{test}$	90.10 (±1.96)	90.82 (±1.87)	90.88 (±1.71)	91.08 (±1.49)	91.55 (±1.33)
*P*	90.10 (±1.95)	90.86 (±1.75)	90.88 (±1.62)	91.07 (±1.41)	91.58 (±1.18)
*R*	90.10 (±1.88)	90.82 (±1.69)	90.88 (±1.57)	91.10 (±1.29)	91.55 (±1.11)
*F*	90.10 (±1.87)	90.81 (±1.66)	90.86 (±1.54)	91.08 (±1.28)	91.55 (±1.09)

## Data Availability

The original contributions presented in this study are included in the article. Further inquiries can be directed to the corresponding author.
